# Supplementary material for: Clathrate Hydrates of Organic Solvents as Auxiliary Intermediates in Pharmaceutical Research and Development: Improving Dissolution Behaviour of a New Anti-Tuberculosis Drug, Perchlozon
Source: Pharmaceutics. 2022 Feb 24;14(3):495. doi: 10.3390/pharmaceutics14030495 (PMC8954777; doi:10.3390/pharmaceutics14030495)
Supplement: Supplementary file 1 [file pharmaceutics-14-00495-s001.zip › pharmaceutics-1554211-supplementary.pdf]

pharmaceutics MDPI

Supplementary Materials: Clathrate Hydrates of Organic Solvents as Auxiliary Intermediates in Pharmaceutical Research and Development: Improving Dissolution Behaviour of a New Anti-Tuberculosis Drug, Perchlozon

Andrey G. Ogienko, Svetlana A. Myz, Andrey A. Nefedov, Anna A. Ogienko, Tatyana P. Adamova, Olga M. Voronkova, Svetlana V. Amosova, Boris A. Trofimov, Vladimir V. Boldyrev and Elena V. Boldyreva

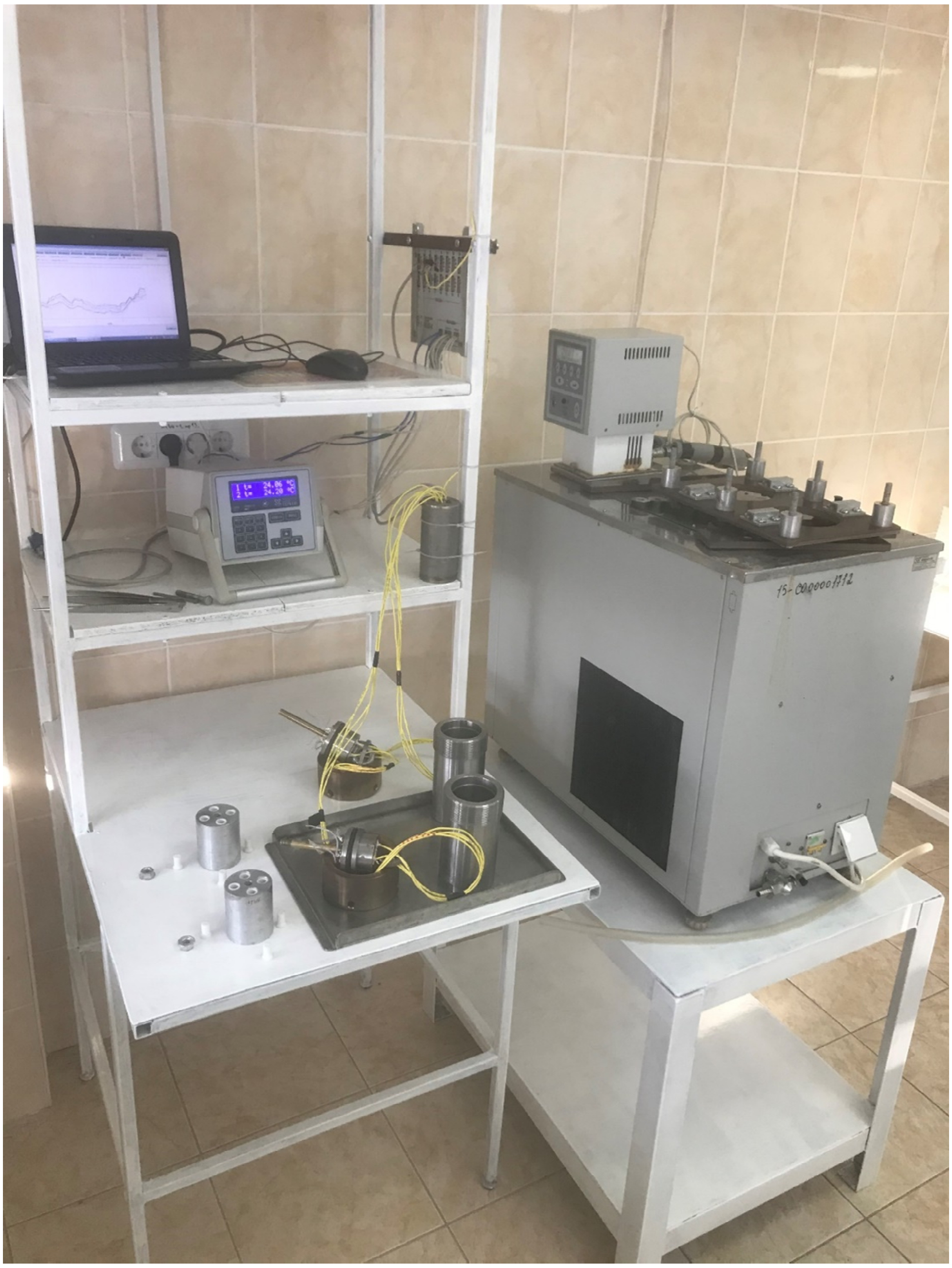

Figure S1. Thermoanalytical (TA) autoclaves before experiments.

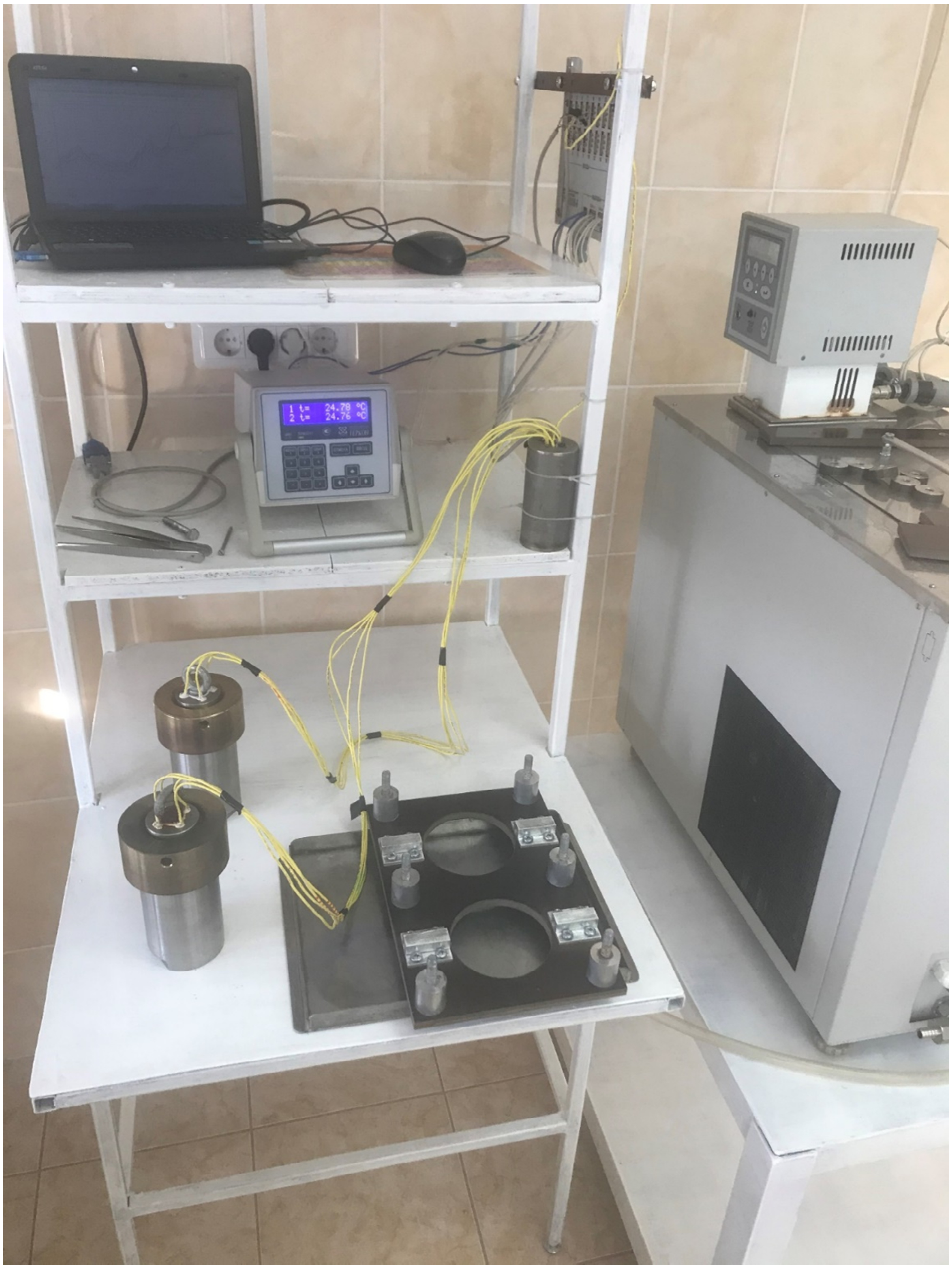

Figure S2. Loaded TA autoclaves.

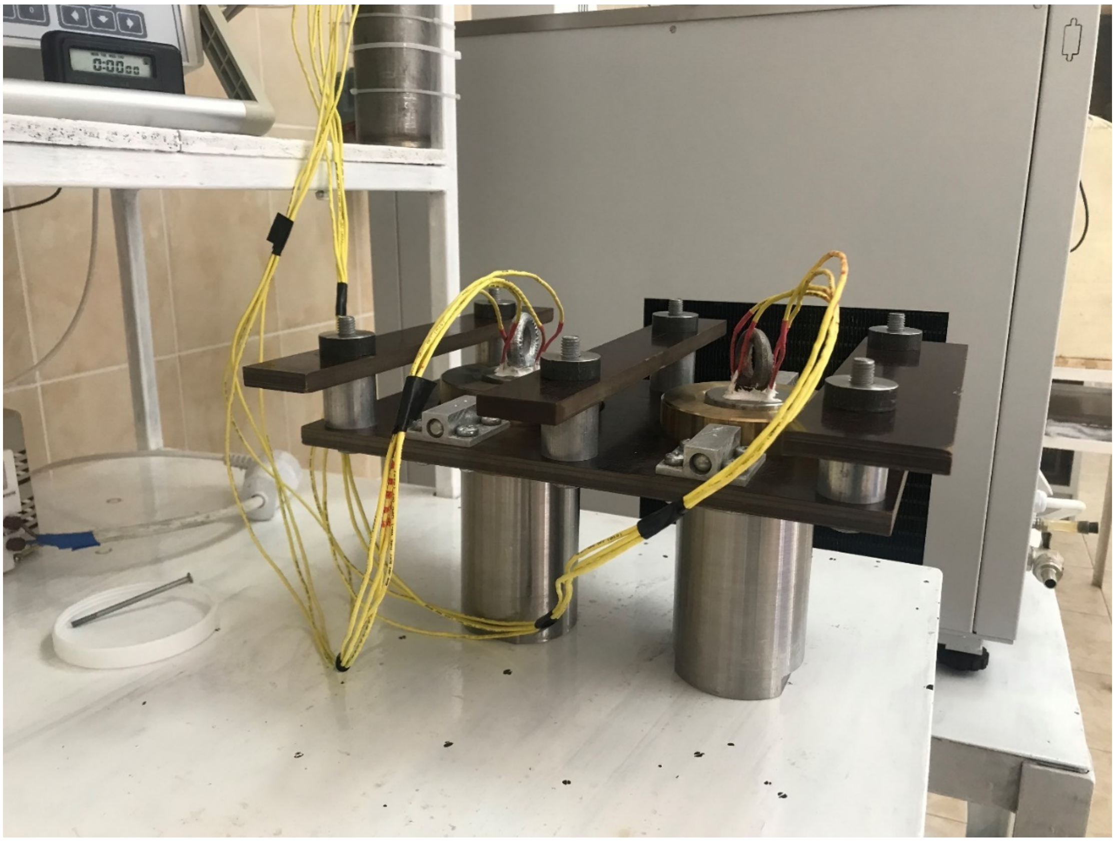

Figure S3. Loaded TA autoclaves in a holder frame, ready to be immersed in a KRIO-BT-06 thermostatic bath.

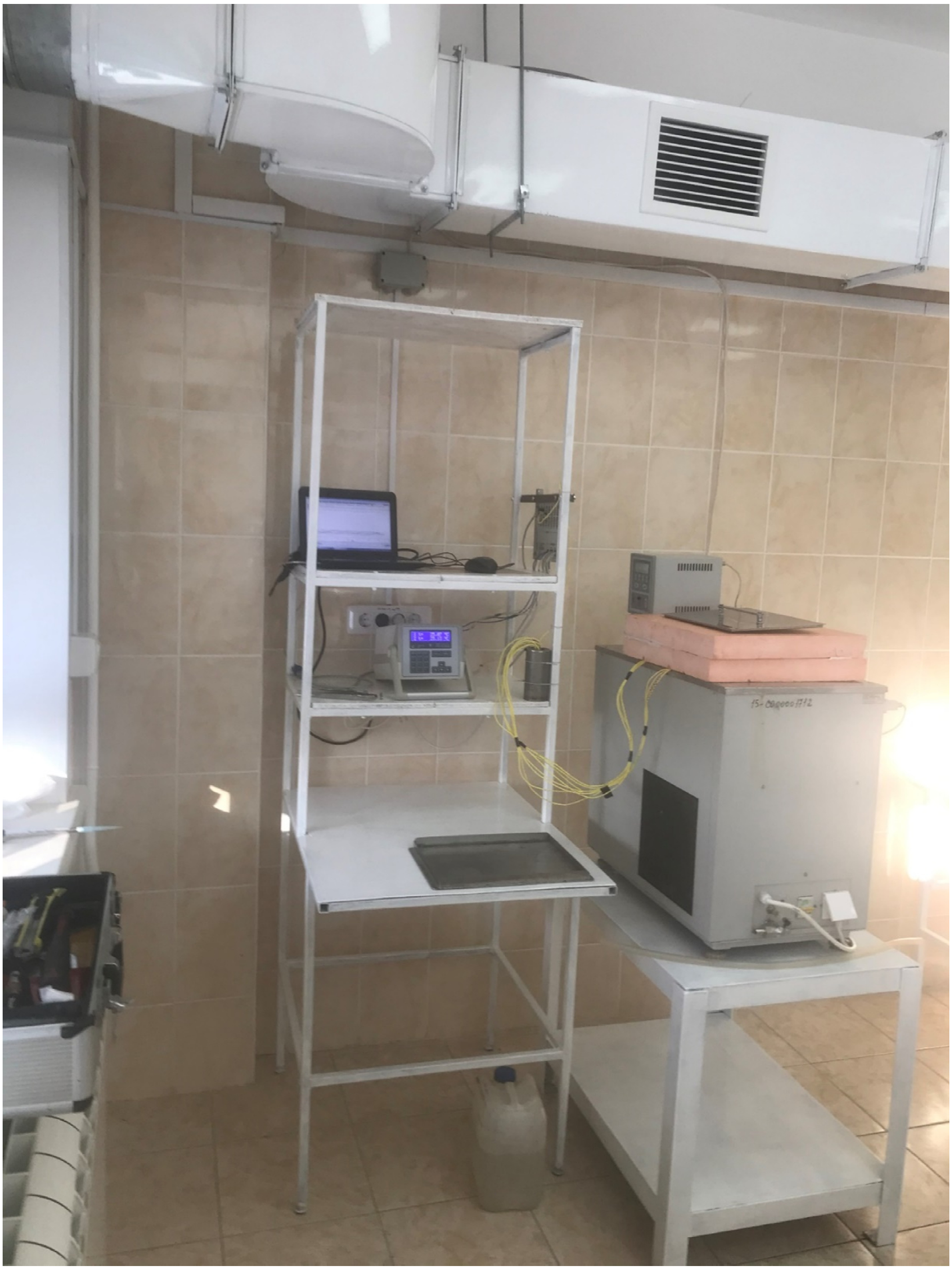

Figure S4. TA autoclaves in a KRIO-BT-06 thermostatic bath. Experiment started.

Table S1. Melting points of Class 3 Residual Solvents and Melting points in some 'Class 3 Solvent – Water' binary systems.

**Solvent**	**Melting point, °C**	**Melting points in 'Class 3 Solvent – Water' binary systems**
tert-Butyl alcohol (TBA)	+26	-3.3°C, 'TBA - hydrate 1' eutectic melting; -8.2°C, 'ice - hydrate 2' eutectic melting [1] -9.3°C, 'ice - hydrate 1'metastable eutectic melting [2]
Acetic acid	+16.6	-26.7°, eutectic melting [3]
Heptane	-90.6	
Acetone	-95	-99°C, 'acetone – hydrate' eutectic melting [4]
Isobutyl acetate	-99	
Anisole	-37.5	
Isopropyl acetate	-73	
1-Butanol	-90.2	
Methyl acetate	-98.1	
2-Butanol	-115	
3-Methyl-1-butanol	-117	
Butyl acetate	-78	
Methyl ethyl ketone	-87	
tert-Butyl methyl ether	-109	
Methyl isobutyl ketone	-80	
Cumene	-96	
2-Methyl-l-propanol	-108	
Dimethylformamide (DMFA)	-61	
Dimethyl sulfoxide (DMSO)	+18.6	-73°C, DMSO·0.5H_2O peritectic melting [5]
Pentane	-130	
Ethanol	-114	-124°C, 'ethanol - hydrate' eutectic melting [6]
1-Pentanol	-78	
Ethyl acetate	-84	
1-Propanol	-127	-168.5°C, '1-propanol - hydrate 1' eutectic melting; -186°C, '1-propanol - hydrate 2' eutectic melting [7]
Ethyl ether	-116	
2-Propanol	-89.5	-98°C, '2-propanol - hydrate' eutectic melting [8]
Ethyl formate	-80	
Propyl acetate	-95	
Formic acid	+8.2	-48.5°, eutectic melting [9]

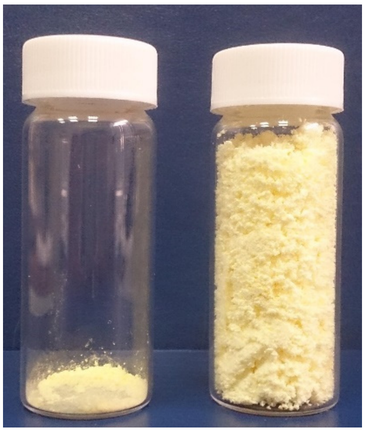

Figure S5. A comparison of the bulk volumes of the same amount (0.145 g) of ultrafine Pz, obtained by Thin Film Freezing, TFF+ Freeze Drying, FD (right) and the starting Pz (left) in 30 ml vials.

This comparison shows that the specific surface area can be increased drastically also for a pure form. However, such an increase is technologically useless. Such powders have zero flowability, they cannot be used, in order to fill in the capsules for capsule DPIs (dry Powder Inhalers). Due to a very low powder density the emitted dose will be extremely low (< 5 mg in Capsule Size "00").

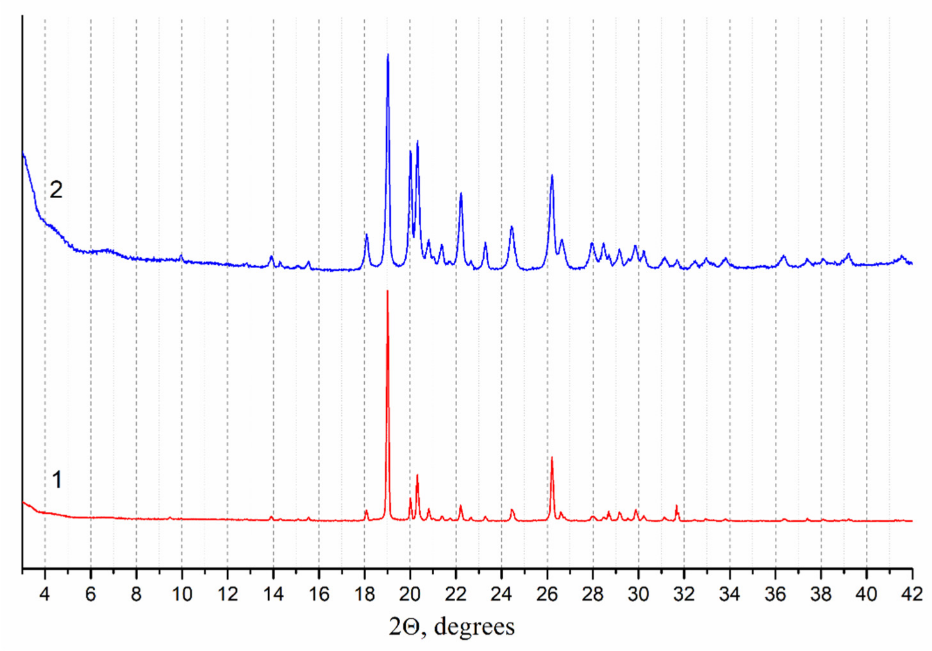

Figure S6. X-ray powder diffraction patterns: (1) starting Pz; (2) ultrafine Pz, obtained by TFF+FD. The sample remains crystalline and has less preferred orientation of the particles.

Calorimetric measurements (experimental details)

Calorimetric experiments were carried out using a DSC 204 F1 Phoenix (Netzsch), in a flow of dry argon (25 ml/min). The drop of the solution (+20°C) was placed into a crucible (which was immediately sealed) and cooled down to -50°C at a cooling rate of 1°/min, and then heated (with measurements from -70°C to 30°C) at the heating rate 3°/min.

The common feature for DSC curves of all frozen benzene solutions is a slope on a baseline in the temperature range ~ −35 ÷ −30°C that may be attributed to a glass transition of the trehalose-containing amorphous freeze-concentrate (Figure S1).

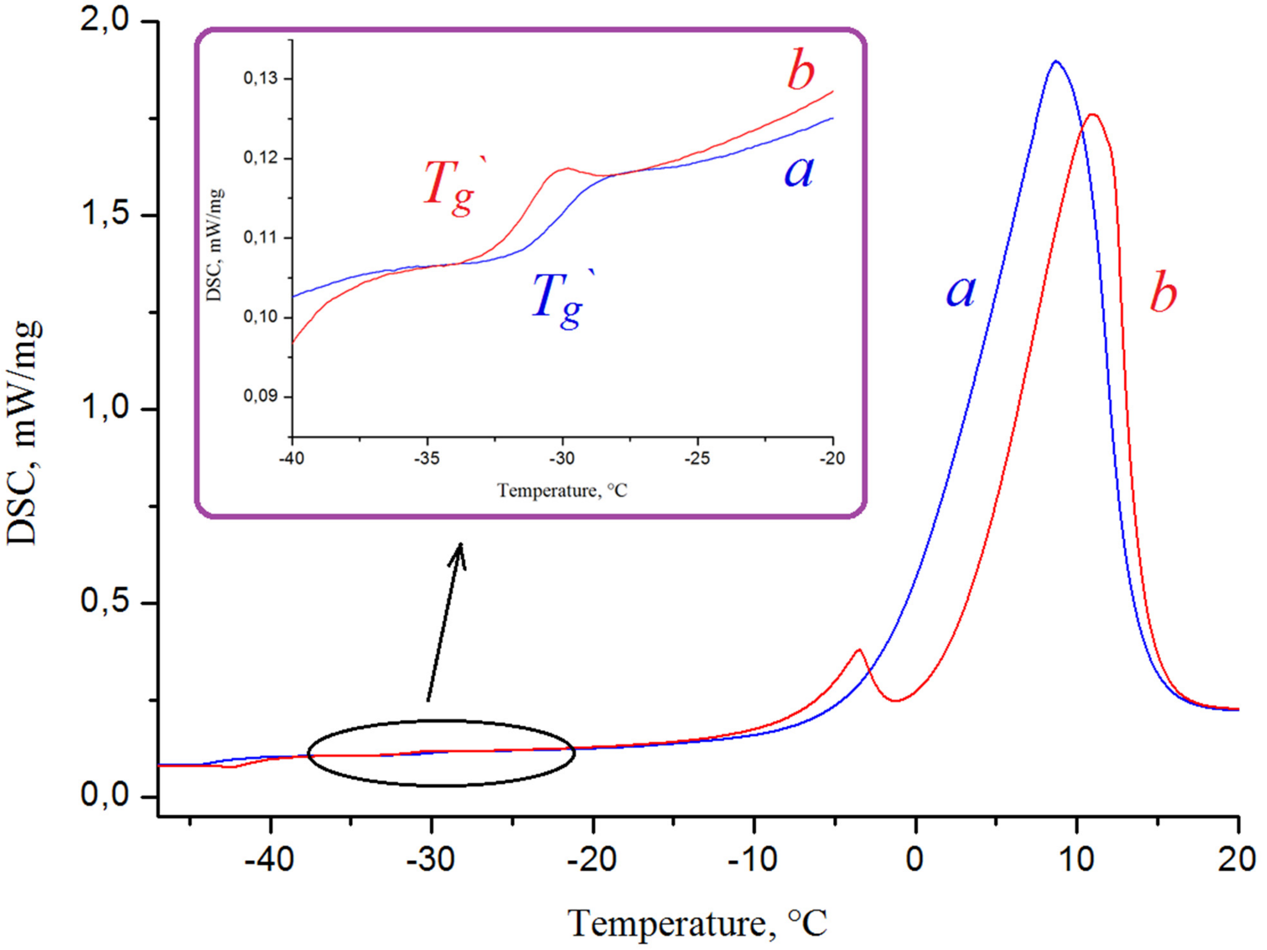

Figure S7. DSC curves of frozen aqueous trehalose solution (*a*) and trehalose solution in THF – water co-solvent system (19.1 wt % of THF) (*b*).

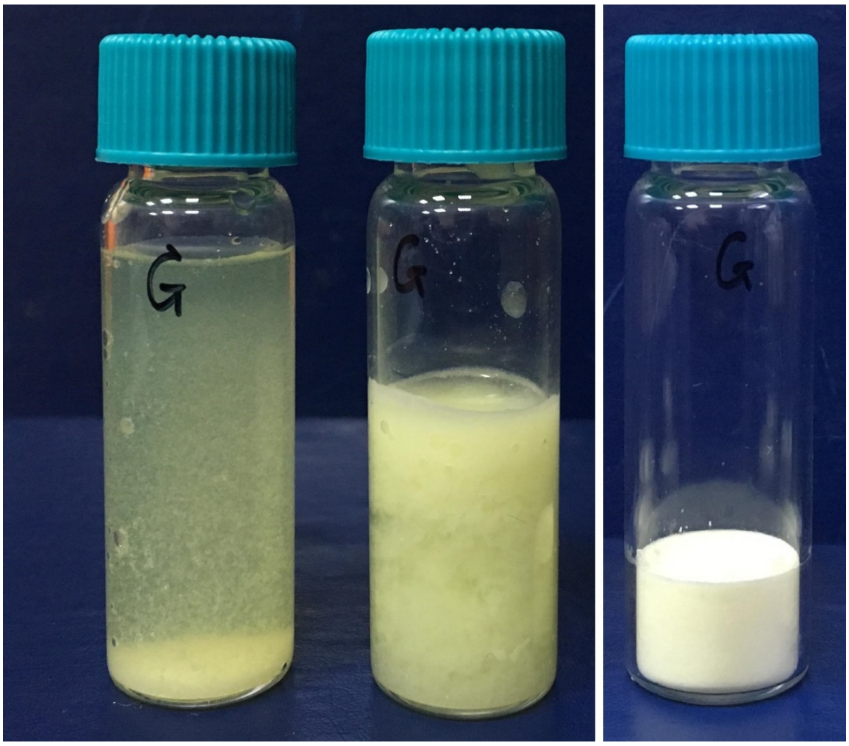

Figure S8. Formation of an unacceptable precipitate on dissolution of Pz formulation with glycine in saline (preliminary experiments). Right to left: starting lyophilized cake (1.00 ml aliquote; 7.75 mg Pz and 31 mg glycine in 1 vial); +3 ml saline; +5 ml saline.

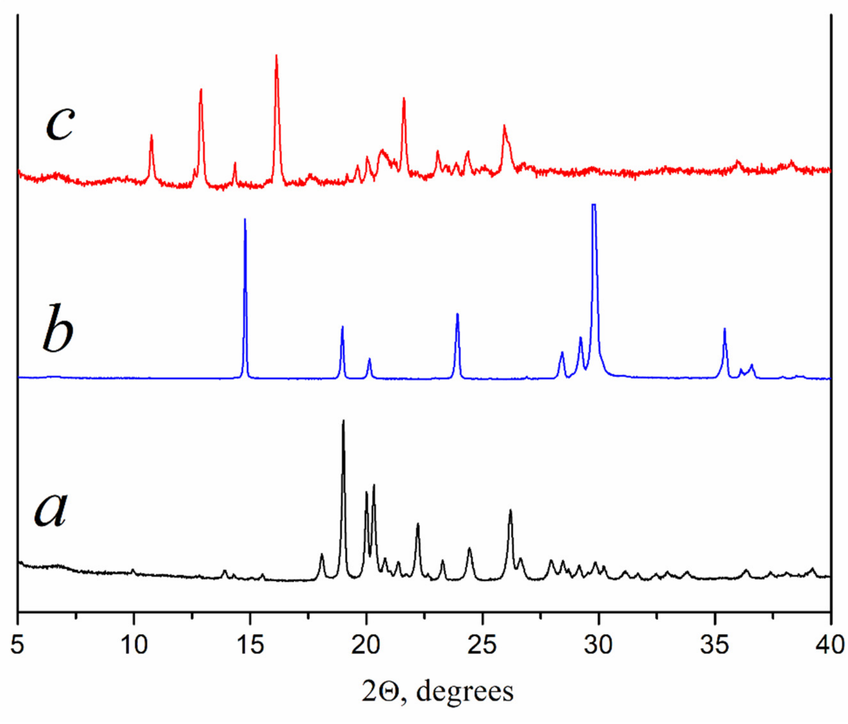

Figure S9. Comparison of X-ray powder diffraction patterns of (*a*) ultrafine Pz, obtained by TFF+FD, (*b*) starting α-glycine and (*c*) precipitate formed on dissolving the Pz formulation with glycine in saline solution.

Table S2. Results of elemental analysis of the precipitate formed on dissolution of Pz formulation with glycine in saline.

Compound	Molecular weight	N, wt %	C, wt %	H, wt %	S, wt %	Cl, wt %
precipitate	236*	23.35	35.60	3.95	13.15	8.0
Pz	280	20.0	30.0	2.9	11.4	-
Pz "base"	180	31.1	46.7	4.4	17.8	-
Pz "base" · Cl^-	215.5	26.0	39.0	3.7	14.9	-
**		0.75	0.76	0.89	0.74	

* Summary of CHNS weight content: 76.05 wt %. ** The ratio of the experimentally found content of C, H, N, S to the calculated one in the Pz "base". Based on the data given above the molecular mass of the precipitate can be estimated as: 180/0.76=236. An estimate of the ratio of the ions Cl^- / ClO_4^- in the precipitate: Define X as the mass ratio of the chloride anion, then

$X*35 + (1-X)*100 = 236-180$

$44 = 65*X$

$X = 0.68$

Therefore, if we do not consider the possibility of the formation of hydrates ($\cdot H_2O$), the formula of the precipitate can be suggested as: **Pz "base" · 0.7 Cl^- · 0.3 ClO_4^-**

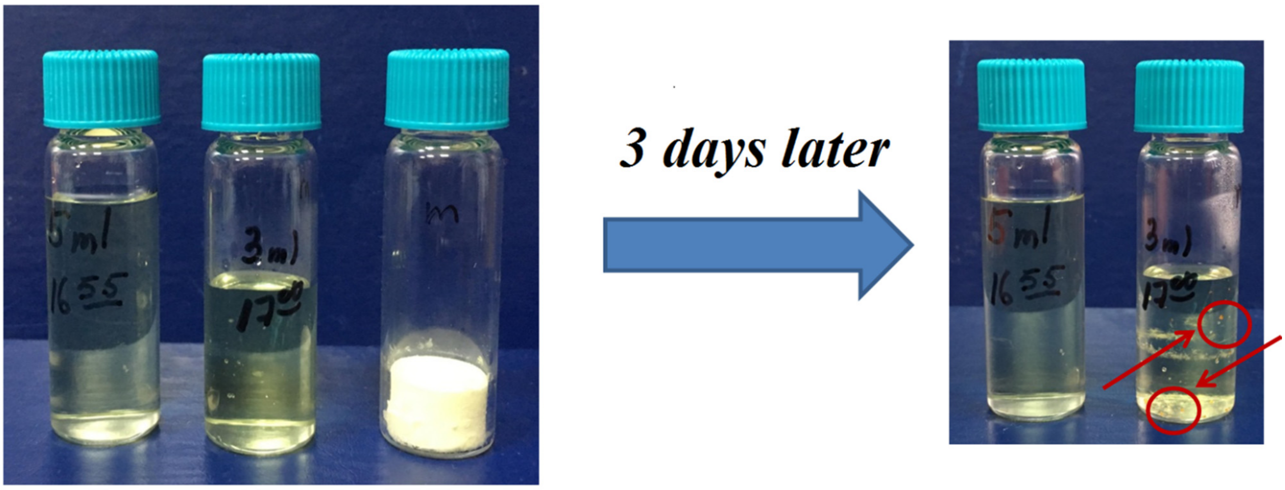

Figure S10. Pz crystallization from saturated saline solution (dissolution of Pz formulation with mannitol (1.00 ml aliquote; 1 vial: Pz, 7.75 mg / mannitol, 31 mg) in 3 or 5 ml of saline). Pz crystals precipitated during storage of the supersaturated solution are pointed by arrows.

References

1. Vessot, S.; Andrieu, J. A review on freeze drying of drugs with tert-butanol (TBA) + water systems: characteristics, advantages, drawbacks, *Dry. Technol*. **2012**, *30*, 377-385. https://doi.org/10.1080/07373937.2011.628133
2. Ogienko, A.G.; Stoporev, A.S.; Ogienko, A.A.; Mel'gunov, M.S.; Adamova, T.P.; Yunoshev, A.S.; Manakov, A.Y.; Boldyreva, E.V. Discrepancy between thermodynamic and kinetic stabilities of the tert-butanol hydrates and its implication for obtaining pharmaceutical powders by freeze-drying, *Chemical Communications* **2019**, *55*, № 29, 4262-4265. https://doi.org/10.1039/C9CC00397E
3. Choi, W.S.; Kim, K. Separation of acetic acid from acetic acid-water mixture by crystallization, J. *Sep. Sci. Technol*. **2013**, *48*, 1056-1061. https://doi.org/10.1080/01496395.2012.718311
4. Dyadin, Y.A.; Bondaryuk, I.V.; Zhurko, F.V. Clathrate hydrates at high pressures. In: Atwood, J.L.; Davies, J.E.D.; MacNicol, D.D., Eds. *Inclusion compounds*. **1991**, *V. 5*. Oxford: Oxford University Press; pp. 214–275.
5. Rasmussen, D.H., MacKenzie, A.P. Phase diagram for the system water–dimethylsulphoxide, *Nature* **1968**, *220*, 1315-1317. https://doi.org/10.1038/2201315a0

6. Zelenin, Y. M. Effect of Pressure on Clathrate Formation in a Water–Ethanol System *J. Struct. Chem*. **2003**, *44*,130-136. https://doi.org/10.1023/A:1024993200295
7. Manakov, A. Y.; Aladko, L. S.; Ogienko, A.G.; Ancharov, A. I. Hydrate formation in the system n-propanol–water, *J. Therm. Anal. Calorim*. **2013**, *111*, 885-890. https://doi.org/10.1007/s10973-012-2246-1
8. Aladko, L. S.; Manakov, A. Y.; Ogienko, A. G.; Ancharov, A. I. New data on phase diagram and clathrate formation in the system water–isopropyl alcohol, *J. Incl. Phenom. Macrocycl. Chem*. **2009**, *63*, 151-157. https://doi.org/10.1007/s10847-008-9499-0
9. Kuznetsova, M. N.; Bergmann, A. G. Physicochemical analysis of the reaction of amines with acids. III. Thermal analysis of the ternary system urea-formic acid-water. *J. General Chem. USSR* **1956**, *26*, 1497–1504; *Chem. Abstr.*, **1956**, *50*, 14337.
